# Doped, conductive SiO_**2**_ nanoparticles for large microwave absorption

**DOI:** 10.1038/s41377-018-0088-8

**Published:** 2018-11-14

**Authors:** Michael Green, Zhanqiang Liu, Peng Xiang, Yan Liu, Minjie Zhou, Xinyu Tan, Fuqiang Huang, Lei Liu, Xiaobo Chen

**Affiliations:** 1 Department of Chemistry, University of Missouri, Kansas City, MO 64110 USA; 2 State Key Laboratory of High Performance Ceramics and Superfine Microstructure, Shanghai Institute of Ceramics, Chinese Academy of Sciences, Shanghai, 200050 China; 3 College of Materials and Chemical Engineering, Hubei Provincial Collaborative Innovation Center for New Energy Microgrid, China Three Gorges University, Yichang, 443002 China; 4 College of Environment, Sichuan Agricultural University, Chengdu, Sichuan 611130 China; 5 School of Chemistry and Chemical Engineering, Hunan Institute of Science and Technology, Yueyang, 414000 China; 6 State Key Laboratory of Luminescence and Applications, Changchun Institute of Optics, Fine Mechanics and Physics, Chinese Academy of Sciences, Changchun, 130033 China

## Abstract

Although many materials have been studied for the purpose of microwave absorption, SiO_2_ has never been reported as a good candidate. In this study, we present for the first time that doped, microwave conductive SiO_2_ nanoparticles can possess an excellent microwave absorbing performance. A large microwave reflection loss (RL) of −55.09 dB can be obtained. The large microwave absorption originates mainly from electrical relaxation rather than the magnetic relaxation of the incoming microwave field. The electrical relaxation is attributed to a large electrical conductivity that is enabled by the incorporation of heterogeneous (N, C and Cl) atoms. The removal of the magnetic susceptibility only results in a negligible influence of the microwave absorption. In contrast, the removal of the heterogeneous atoms leads to a large decrease in the electrical conductivity and microwave absorption performance. Meanwhile, the microwave absorption characteristics can be largely adjusted with a change of the thickness, which provides large flexibility for various microwave absorption applications.

## Nanoscience: microwave absorption

Doped nanoparticles of silica (SiO_2_) have been found to act as a very strong absorber of microwave radiation. A US-Chinese collaboration of scientists discovered that, unlike pure SiO_2_ nanoparticles, those doped with atoms of N, C and Cl induce a reflection loss at large as −55 dB at a frequency of around 7 GHz. The doped nanoparticles, which measured 4–8 nm in diameter, were fabricated by slowly adding the precursor tetraethyl orthosilicate (TEOS) to the solvent *N*,*N*’-dimethylformamide (DMF) and then adding hydrazine monohydrochlorid. The resulting solution was then heated, washed and dried. The resulting nanoparticles were then dispersed in paraffin wax rings and the permittivity and permeability measured in the 1.0–18.0 GHz range using a network analyzer. The team attributes the strong microwave absorption to the doped nanoparticles’ good electrical conductivity.

## Introduction

The utility of microwave absorption is significant in many applications, such as wireless communications and anti-radar detection of aircraft^1–3^. For example, many materials have been developed to reduce the radar signature of aircraft, ships and tanks in military fields. Material examples include carboneous materials such as graphite^4^, graphene^5^, carbon nanotubes (CNTs)^6^ and carbon fibers^7^, conducting polymers^8^, oxides such as Fe_2_O_3_^9^, Fe_3_O_4_^10^, MnO_2_^11^, ZnO^12^, BaFe_12_O_19_^13^, BaTiO_3_^13^, SrFe_12_O_19_^14^, and carbides such as SiC^15^ and SiCN^16^. Traditional mechanisms that are commonly believed to be responsible include dipole rotation and magnetic domain resonance due to the dielectric and magnetic losses inside the materials. For example, the microwave absorption of Ni-coated CNT/epoxy composites results from dielectric and magnetic losses, with the use of Ag nanowires shown to enhance the performance due to dielectric loss^3^. Conducting polymers such as polypyrrole, polyaniline and poly(3-octylthiophene) have been demonstrated to show good microwave absorption^8^. Suitable inclusion of Fe_2_O_3_ nanoparticles in polyaniline enhanced the microwave absorption properties due to simultaneous adjustment of dielectric loss and magnetic loss^9^. Hollow urchin-like MnO_2_ nanostructures with tetragonal nanorod clusters showed better performance due to proper electromagnetic impedance matching^11^. BaTiO_3_/polyaniline and BaFe_12_O_19_/polyaniline composites showed good compatible dielectric and magnetic properties for broadband microwave absorbing properties^13^. Recently, microwave absorption has been reported to be enhanced by defective or disorder structures such as oxygen vacancies, low crystallinity and use of a core/shell structure^1,2,17–19^. The dielectric properties of TiO_2_^1,2,17,18^, ZnO^2^ and BaTiO_3_^19^ nanoparticles can be modified through the perturbation of their crystalline structure by hydrogenation treatment to enhance their microwave absorption performance. Gao and colleagues^20^ have also found that the microwave dielectric properties of TiO_2_ nanoparticles can be largely altered by introducing partial crystalline phases in such TiO_2_ nanoparticles. Although these findings have provided a large pool of materials for microwave absorption applications, each material may have some advantages and disadvantages; therefore, it is highly desirable to discover new materials for microwave absorption. On the other hand, microwave absorption is also very useful for sample preparation with microwave irradiation in that rapid synthesis is enabled when compared to use of a traditional heating treatment^21,22^.

Up to now, pure SiO_2_ has not yet been reported to show a good microwave absorption performance, although an enhanced microwave performance for the composites of SiO_2_/carbon^23^, SiO_2_/carbonyl iron^24^ and SiO_2_/iron^25^ was obtained due to the combination of a proper electromagnetic impedance match^23–25^. This is understandable because in pure SiO_2_ there is no good source for traditional microwave absorption mechanisms including dipole rotation and magnetic domain resonance due to dielectric and magnetic losses. The lack of origin for the creation of dipoles in pure SiO_2_ nanoparticles is attributed to its symmetric structure where the dipoles are canceled in each tetrahedral unit of the SiO_2_ lattice. Meanwhile, it is also unlikely that there will be magnetic domains to echo with the microwave electromagnetic field due to the lack of charge spin centers in pure SiO_2_. Therefore, it is reasonable that pure SiO_2_ will not be able to possess good mechanisms for efficient microwave absorption. To enable effective microwave absorption, we intentionally create dipoles in SiO_2_ nanoparticles by introducing heterogeneous atoms such as C, N and Cl elements on the O sites inside the SiO_4_ tetrahedra or simply linked onto the surface of the SiO_2_ nanoparticles. Rotations of these induced dipoles can, therefore, echo with the incident microwave field to possess microwave absorption. Effective electrical loss can be achieved with such heterogeneous atoms by having higher electrical conductivity. Here, we report that the doped, microwave conductive SiO_2_ nanoparticles can show excellent microwave absorption performance. In contrast, only poor microwave absorption performance is observed for the SiO_2_ nanoparticles without the incorporation of the heterogeneous atoms, which lack dipole rotations and good electrical conductivity.

## Materials and methods

Tetraethyl orthosilicate (TEOS), *N*,*N*’-dimethylformamide (DMF) and hydrazine monohydrochloride were purchased from Sigma-Aldrich and used as received. Typically, TEOS (2.2 mL) was slowly added to DMF (20.0 mL) under ambient conditions by vigorously stirring, producing a completely transparent solution. A desired amount of hydrazine monohydrochloride (2.74 g) was added and stirred for 5–10 min, which was then transferred into a Teflon-lined, stainless autoclave at a different temperature of 160 °C for 12 h, producing a light-yellow slurry, which was washed with anhydrous ethanol and dried at 100 °C. A light-yellow product was collected. For comparison, pure white SiO_2_ nanoparticles were also prepared followed by calcination of the light-yellow sample at 600 °C in air for 2 h.

The crystal structure was examined using a Rigaku Miniflex X-ray diffractometer (XRD) with a Cu Kα (λ = 0.15418 nm) radiation source. The morphology and crystallinity were probed with transmission electron microscopy (TEM) and high-resolution TEM (HRTEM) on an FEI Tecnai F20 STEM under an electron accelerating voltage of 200 kV. The surface chemical properties were studied with a Kratos Axis 165 X-ray photoelectron spectrometer (XPS) equipped with an Al/Mg dual-anode X-ray source. All the XPS spectra were calibrated with the C 1s peak from the carbon tape to 284.6 eV. The energy-dispersive X-ray (EDX) spectrum was taken using a Tescan Vega 3 LMU scanning electron microscope equipped with a Bruker Quantax 6|10 EDX system. The Fourier transform infrared (FT-IR) spectra were collected using a Thermo-Nicolet iS10 FT-IR spectrometer equipped with an attenuated total reflectance unit. The complex permittivity and permeability were measured in the frequency range of 1.0–18.0 GHz using an HP8722ES network analyzer with ring-shape samples containing 60 wt% SiO_2_ nanoparticles dispersed in paraffin wax. The ring had a thickness of 2.0 mm, an inner diameter of 3.0 mm and an outer diameter of 7.0 mm.

## Results and discussion

**Fig. 1:**
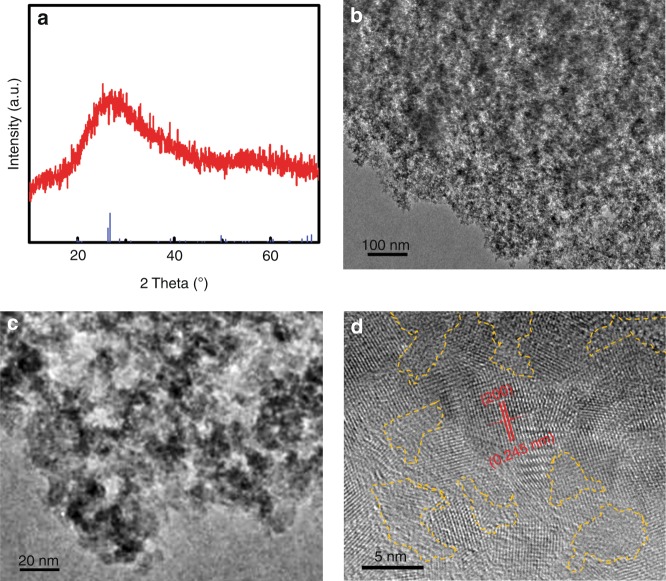
**Physical properties of SiO**_**2**_ **nanoparticles. a** XRD pattern, **b**, **c** TEM and **d** HRTEM images of SiO_2_ nanoparticles. The panel (**a**) also shows the standard (PDF#00-038-0360). The yellow dashed lines in (**d**) point out the amorphous phases

The XRD pattern for the doped SiO_2_ nanoparticles matched well with the standard card of SiO_2_ (PDF#00-038-0360), as shown in Fig. 1b. The broadness of the broad peak at approximately 26.2° indicated that the nanoparticle size was small. The weak intensity suggested a poor crystallinity. From the TEM image in Fig. 1c, clearly, the nanoparticles were 4–8 nm in diameter (Fig. 1d). The HRTEM image in Fig. 1e exhibited clear lattice fringes with a plane distance of 0.245 nm corresponding to the (200) plane of monoclinic Moganite SiO_2_. Meanwhile, some amorphous regions were also observed between the crystalline domains, suggesting the likely existence of an amorphous phase (as pointed out by the dashed lines). The small size and amorphous phase matched well with the broad diffraction peak from the XRD pattern. This coexistence of amorphous and crystalline domains in the doped SiO_2_ nanoparticles was similar to that reported for TiO_2_ nanoparticles showing enhanced microwave absorption;^1,2,20^ this is because the amorphous phase may create some interfacial dipole rotations along the interface with the crystalline phase and induce active microwave absorption^1,2,20^. The existence of Si, O, C, N and Cl elements were confirmed using results from XPS and EDX, as shown in Figure S1-S7. The surface of the doped SiO_2_ nanoparticles was likely linked with the –NH_2_ groups from the hydrazine hydrochloride and some adsorbed water and HCl molecules (Figure S8). The Si 2*p* spectrum (Figure S2) showed one peak with a binding energy centered approximately 103.2 eV, corresponding to the Si 2*p*_3/2_ in the SiO_2_^26,27^. In the O 1s XPS spectrum shown in Figure S3, one peak was found to be centered approximately 532.6 eV, consistent with the binding energy of the O 1s signal from SiO_2_^28,29^. The C 1s XPS spectrum (Figure S4) showed one minor peak centered approximately 284.6 eV, and one major peak approximately 286.4 eV. The former was likely from the adsorbed carbon during the XPS measurement, with the latter likely from the alkyl groups from the TEOS on the surface of the SiO_2_ nanoparticles^30,31^. The N 1s spectrum in Figure S5 showed two peaks with a major contribution approximately 399.9 eV and a minor shoulder approximately 401.9 eV, likely from the N_2_H_4_ moiety and the conjugated NH_2_ moiety attached to the HCl group from the hydrazine hydrochloride on the surface of the SiO_2_ nanoparticles, respectively^32,33^. The Cl 2*p* XPS spectrum (Figure S6) displayed one higher peak approximately 197.6 eV and one lower peak near 199.3 eV, likely from the HCl coupled to the N_2_H_4_ moiety on the surface and the HCl coupled to the SiO_2_ surface, respectively^34^.

**Fig. 2:**
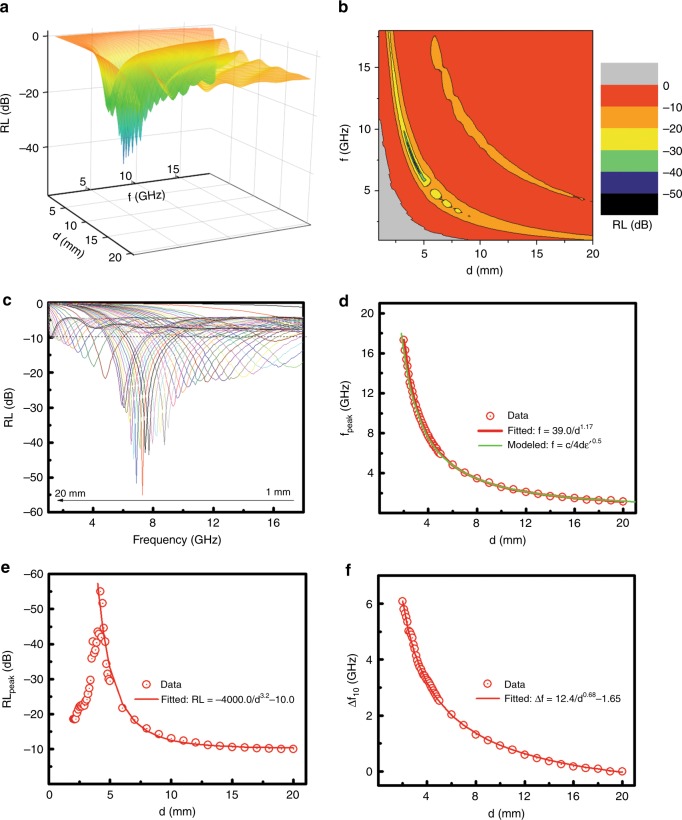
**Microwave absorption characteristics of SiO**_**2**_ **nanoparticles. a** The 3D plot and **b** 2D contour of the RL curves with *d* and *f*, **c** the RL curves, **b** the relationship for the **d** *f*_peak_, **c**, **e** RL_peak_ and **d**, **f** Δ*f*_10_ with *d* of the SiO_2_ nanoparticles in the frequency range of 1.0–18.0 GHz

The variations of the microwave reflection loss (RL) with frequency (*f*) and thickness (*d*) were clearly displayed in a three-dimensional (3D) plot and two-dimensional (2D) contour, as shown in Fig. 2a, b. The 2D contour plot showed the projection of the 3D graph for the change of RL (indicated by the color ruler) on the frequency *f* and thickness *d* plane. The different colors indicated where (*f* and *d*) and which level of RL was achieved. Some representative RL curves were shown in Fig. 2c for varying *d* from 1.0 to 20.0 mm. The maximum microwave absorption frequency (*f*_max_) was tunable with *d*. As *d* increased, *f*_peak_ shifted from higher frequency to lower frequency, as clearly seen in Fig. 2d. The relationship between *f*_peak_ and *d* was fitted very well with the formula *f*/GHz = 39.0/(*d*/mm)^1.17^ or *c*/4*f*_peak_ε’^0.5^ = λ/4ε’^0.5^, where *c* was the speed of light. Apparently, *f*_peak_ decreased reversibly with *d*. As *d* became bigger, *f*_peak_ decreased. The change in RL_peak_ with *d* (Fig. 2e) could be divided into two stages: a quick increase from −18.45 to −55.09 dB when *d* was changed from 2.0 to 4.2 mm, and a decay from −55.09 to −9.97 dB as *d* grew from 4.2 to 20.0 mm. The largest RL_max_ value (−55.09 dB) was observed when *d* was 4.2 mm. The change in Δ*f*_10_ with *d* was shown in Fig. 2f and fitted very well with Δ*f*_10_ /GHz = 12.4/(*d*/mm)^0.68^ – 1.65. As *d* became bigger, Δ*f*_10_ almost decreased monotonically. This indicated that a thicker coating actually shielded a narrower region of microwave reflection frequency. It should be noted that the application of a thicker coating is very useful for the protection of important stationary objects on the ground from radar detection where a thin coating may not be able to shield such objects at a specific frequency, which is very important but frequently overlooked.

**Fig. 3:**
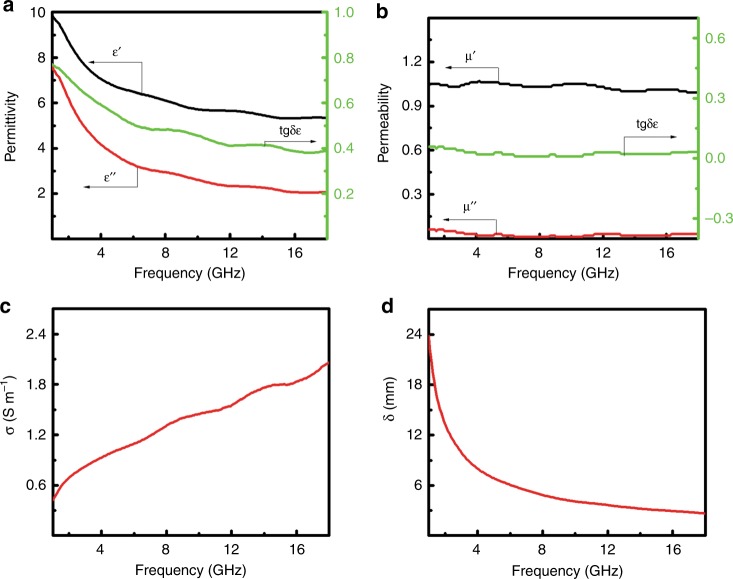
**The dielectric and magnetic properties of SiO**_2_ **nanoparticles. a** The complex permittivity (*ε*’, *ε*”, *tgδ*_ε_), **b** complex permeability (*μ*’, *μ*”, *tgδ*_μ_), **c ** electrical conductivity (*σ*) and **d** skin depth (*δ*) of the SiO_2_ nanoparticles in the frequency range of 1.0–18.0 GHz

The microwave absorption properties are dependent on the dielectric and magnetic properties:

1 $${\mathrm{RL}}\left( {{\mathrm{dB}}} \right) = 20log \left| {\left( {Z_{{\mathrm{in}}}- Z_{\mathrm{0}}} \right){\mathrm{/}}\left( {Z_{{\mathrm{in}}} + Z_{\mathrm{0}}} \right)} \right|$$

2 $${Z}_{{\mathrm{in}}} = Z_{\mathrm{0}}(\mu_{\mathrm{r}}/\varepsilon _{\mathrm{r}})^{1/2}{\mathrm{tanh}}\left[ {j(2\pi fd/c)(\varepsilon _{\mathrm{r}}\mu _{\mathrm{r}})^{{1/2}}} \right]$$

where RL(dB) is the reflection loss in dB, *Z*_in_ is the input impedance of the absorber, *Z*_0_ is the impedance of free space, *μ*_r_ is the relative complex permeability, *ε*_r_ is the relative complex permittivity, *f* is the frequency of the electromagnetic wave, *d* is the thickness of the absorber and *c* is the velocity of light^18^. As shown in Fig. 3a, when *f* increased from 1.0 to 18.0 GHz, *ε*’ gradually decreased from 9.89 to 5.35, *ε*” decreased slowly from 7.63 to 2.06 and *tgδ*_ε_ fell slowly from 0.77 to 0.39. Figure 3b showed that *μ*’ decreased from 1.05 to 0.99, *μ*” dropped from 0.06 to 0.03 and *tgδ*_μ_ changed from 0.06 to 0.03 when *f* increased from 1.0 to 18.0 GHz. These results suggested that the doped SiO_2_ nanoparticles had a smaller stored electrical and magnetic energy as the frequency of the incident electromagnetic field increased, indicating that some of the echoes of the electric field or dipoles to the oscillating field lagged behind and seemed consumed as the frequency increased.

The electrical conductivity (*σ*) shown in Fig. 3c was calculated with *σ* (S m^−1^) *=* *2πfε*_*0*_*ε”*, where *ε*_0_ was the free space permittivity (8.854 × 10^−12^ F m^−1^), *f* was the frequency (Hz) and *ε*” was the imaginary component of the permittivity^35^. The *σ* increased almost monotonically from 0.42 to 2.06 S m^−1^ as *f* increased from 1.0 to 18.0 GHz. The large conductivity was suggested to be possibly related to the existence of the heterogeneous atoms (C, N, Cl) on the surface of the SiO_2_ nanoparticles; for example, partial oxygen atoms were replaced with N atoms on the surface of the particles. The skin depth (*δ*) of the microwave irradiation in Fig. 3d was calculated with (δ/m) = (*πfμ*_0_*μ*_r_*σ*)^−1/2^, where *μ*_0_ was the permeability of free space (4π × 10^−7^ H m^−1^), *μ*_r_ was the relative permeability and *σ* was the electrical conductivity (S m^−1^)^33^. The *δ* decreased from 23.84 to 2.63 mm as *f* increased from 1.0 to 18.0 GHz.

**Fig. 4:**
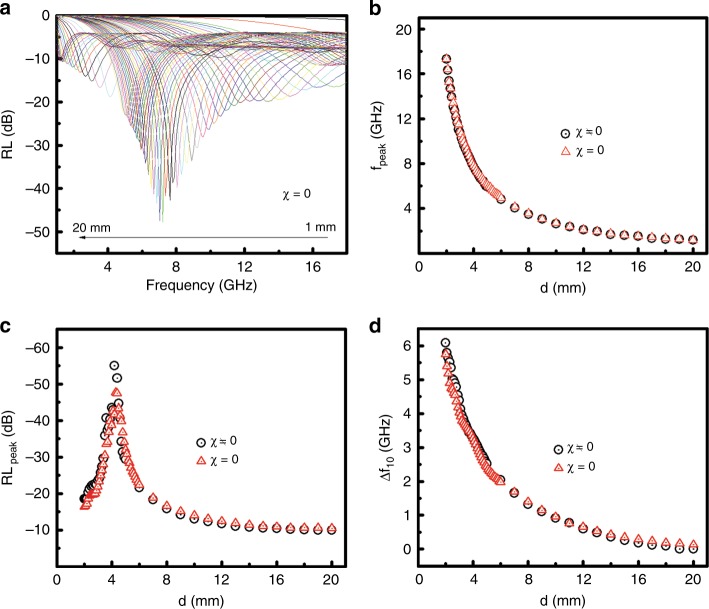
**Some microwave absorption characteristics of SiO**_**2**_ **nanoparticles with/out magnetic contribution. a** The RL curves when *χ*_m_ is zero, and a comparison of the relationships for the **b** *f*_peak_, **c** RL_peak_ and **d** Δ*f*_10_ with *d*, when *χ*_m_ is nonzero vs. zero for the SiO_2_ nanoparticles in the frequency range of 1.0–18.0 GHz

To reveal the contribution of the permeability or the magnetic properties of the SiO_2_ nanoparticles to the microwave absorption performance, we compared the microwave absorption results with and without the contribution of the magnetic components by assuming, for the latter case, a magnetic susceptibility parameter (*χ*_m_) equal to zero, where *μ*_r_ = *μ*/*μ*_0_ = (1 + *χ*_m_)*μ*_0_. The evolution of the RL curves in Fig. 4a when *d* was changed from 1.0 to 20.0 mm indicated that as *d* increased, *f*_peak_ shifted to lower values. As shown in Fig. 4b, *f*_peak_ decreased as *d* increased, with their relationships overlapping almost completely for nonzero and zero *χ*_m_ (see also Figure S9). RL_peak_ rapidly increased and then decayed with *d*, following the same trend for nonzero *χ*_m_ (Fig. 4c and Figure S10). Meanwhile, it was noticeable that the maximum RL_peak_ was smaller with a zero *χ*_m_ as *d* changed. Figure 4d compared the Δ*f*_10_–*d* relationships for nonzero and zero *χ*_m_. The Δ*f*_10_–*d* trends overlapped very well (also see Figure S11) despite Δ*f*_10_ being smaller at smaller *d* values and larger at larger *d* values for zero *χ*_m_. This indicated that the influence of the none-zero *χ*_m_ for the SiO_2_ nanoparticles was mainly reflected in the change of the Δ*f*_10_ values. Overall, the none-zero *χ*_m_ increased the RL_peak_ and the Δ*f*_10_ values possibly achieved at certain *d* values despite the overall impact being small. The small contribution of the magnetic property on the microwave absorption was related to the small *μ*” and *tgδ*_μ_ values. The overall influence of the none-zero *χ*_m_ on the microwave absorption was clearly shown in Figure S12 as well.

Therefore, the large microwave absorption performance of the doped, conductive SiO_2_ nanoparticles was most likely related to their dielectric properties, or the rotations of dipoles in the material as indicated by the large electrical conductivity in the microwave frequency range, as shown in Fig. 3c. In pure SiO_2_ nanoparticles, no obvious origin was found for the creation of dipoles due to its symmetric structure where the dipoles likely canceled out each other in each tetrahedral unit of the SiO_2_. However, in the doped SiO_2_ nanoparticles, there were possible sources for the existence of dipoles due to the introduction of C, N and Cl elements as evidenced by the XPS results. Those atoms apparently broke down the symmetrical environment of the SiO_2_ lattice, and created dipoles on the surface, causing the variation of the dielectric property across the nanoparticles and the increased conductivity. Under microwave irradiation, those dipoles might rotate and echo with the electromagnetic field to produce resonance, causing reflection loss, as schematically shown in Figure S13. Therefore, a large RL was observed in the case of a good match between the dielectric/magnetic properties with the incident microwave field.

**Fig. 5:**
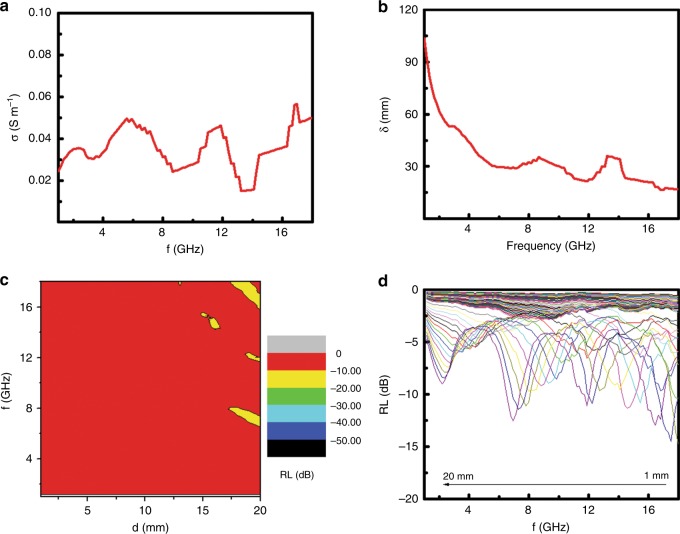
**Some dielectric and microwave absorption characteristics of calcinated, undoped SiO**_**2**_ **nanoparticles. a** The *σ* curve, **b** *δ* curve, **c** 2D contour plot for RL vs. *f* and *d* and **d** representative RL curves for the pure, poorly conductive SiO_2_ nanoparticles obtained after calcination

To verify this conclusion, we measured the microwave absorption performance along with the dielectric and magnetic properties of those doped SiO_2_ nanoparticles after removal of the heterogeneous atoms by calcination at 600 °C for 2 h in air. The removal of those atoms was confirmed using XPS results (Figure S14-17) where no N and Cl atoms were observed, with the remaining C due to atmospheric deposition. Meanwhile, calcination at high temperature normally led to high crystallization and removal of the amorphous phase in the material. Compared to the doped SiO_2_ nanoparticles, the calcinated, pure SiO_2_ nanoparticles showed much smaller *ε*’, *ε*” and *tgδ*_ε_ values (Figure S18), which matched well with the values in the literature^36^, but showed similar *μ*’, much larger *μ*” and *tgδ*_μ_ values (Figure S19). As a result, these calcinated, pure SiO_2_ nanoparticles only showed very small *σ* values (Fig. 5a) and large *δ* (Fig. 5b) values in the microwave region. The small *σ* values indicated that these SiO_2_ nanoparticles were barely electrically conductive, with the large *δ* values suggesting that the microwave field was not efficiently decayed. Their poor microwave absorption performance was observed from the 2D contour plot of the RL over *f* and *d* in Fig. 5c and the RL plots in Fig. 5d, where the RL values were found to be less than −10 dB in most of the *d*–*f* regions. The poor microwave absorption performance of the calcinated SiO_2_ nanoparticles further confirmed that the good microwave absorption performance of the doped SiO_2_ nanoparticles was due to electrical relaxation, as the calcinated SiO_2_ nanoparticles had much smaller electrical relaxation but much large magnetic relaxation. Meanwhile, the fact that the associated C, N and Cl atoms were removed from the SiO_2_ nanoparticles after calcination also hinted that those heterogeneous atoms were likely linked onto the surface instead of being located in the bulk of the SiO_2_ nanoparticles. Table S1 listed the microwave absorption performance of various materials that have been studied. As seen, doped, microwave conductive SiO_2_ showed an impressive microwave absorption performance. Therefore, such materials are a promising candidate for microwave absorption. The importance of electrical relaxation on the microwave absorption is consistent with the conclusion made in the studies by Mao and colleagues^37–43^. Furthermore, such materials can be shown to be very useful in self-powered electromagnetic energy conversion and microwave attenuation, as demonstrated by Mao and colleagues^40,41^, which we plan to build in our future work.

## Conclusions

In summary, in this study we have shown that doped, microwave conductive SiO_2_ nanoparticles possess an exciting microwave absorbing performance, benefiting from the good electrical conductivity that is induced by the incorporation of heterogeneous (N, C and Cl) atoms on the surface of the SiO_2_ nanoparticles. A large RL value of −55.09 dB can be obtained. The microwave absorption is mainly related to the dielectric loss resulting from the good electrical conductivity, while the small contribution of the magnetic property may be directly related to the small *μ*” and *tgδ*_μ_. In contrast, calcinated, pure SiO_2_ nanoparticles show a poor electrical conductivity and microwave absorption performance even with a larger magnetic response. In addition, microwave absorption characteristics such as *f*_peak_, RL_peak_ and Δ*f*_10_ can be largely adjusted with the thickness of the doped, conductive SiO_2_ nanoparticles, which provides large flexibility for their microwave application towards various purposes.

## Electronic supplementary material

Supplemental materials
