# Supplementary material for: Doped, conductive SiO2 nanoparticles for large microwave absorption
Source: Light Sci Appl. 2018 Nov 14;7:87. doi: 10.1038/s41377-018-0088-8 (PMC6234207; doi:10.1038/s41377-018-0088-8)
Supplement: Supplementary file 1 — Supplemental materials [file 41377_2018_88_MOESM1_ESM.docx]

**Supplemental Material**

**Doped, conductive SiO_2_ nanoparticles for large microwave absorption**

Michael Green,^1^ Zhanqiang Liu,^2^ Peng Xiang,^3^ Yan Liu,^1,4^ Minjie Zhou,^1,5,*^ Xinyu Tan,^3,*^ Fuqiang Huang,^2*^ Lei Liu,^6,*^ Xiaobo Chen^1,*^

^1^Department of Chemistry, University of Missouri – Kansas City, Missouri, 64110, USA.

^2^State Key Laboratory of High Performance Ceramics and Superﬁne Microstructure, Shanghai Institute of Ceramics, Chinese Academy of Sciences, Shanghai, 200050, China.

^3^College of Materials and Chemical Engineering, Hubei Provincial Collaborative Innovation Center for New Energy Microgrid, China Three Gorges University, Yichang, 443002, China.

^4^College of Environment, Sichuan Agricultural University, Chengdu, Sichuan, 611130, China.

^5^School of Chemistry and Chemical Engineering, Hunan Institute of Science and Technology, Yueyang, 414000, China.

^6^State Key Laboratory of Luminescence and Applications, Changchun Institute of Optics, Fine Mechanics and Physics, Chinese Academy of Sciences, Changchun, 130033, China.

Email: zmj0104@163.com; tanxin@ctgu.edu.cn; huangfq@mail.sic.ac.cn; liulei@ciomp.ac.cn; chenxiaobo@umkc.edu.

**Figure S1-S19, Table S1.**

**Figure S1.** Survey XPS spectrum of the doped, conductive SiO_2_ nanoparticles.

**Figure S2.** Si 2p core-level XPS spectrum of the doped, conductive SiO_2_ nanoparticles.

**Figure S3.** O 1s core-level XPS spectrum of the doped, conductive SiO_2_ nanoparticles.

**Figure S4.** C 1s core-level XPS spectrum of the doped, conductive SiO_2_ nanoparticles.

**Figure S5.** N 1s core-level XPS spectrum of the doped, conductive SiO_2_ nanoparticles.

**Figure S6.** Cl 2p core-level XPS spectrum of the doped, conductive SiO_2_ nanoparticles.


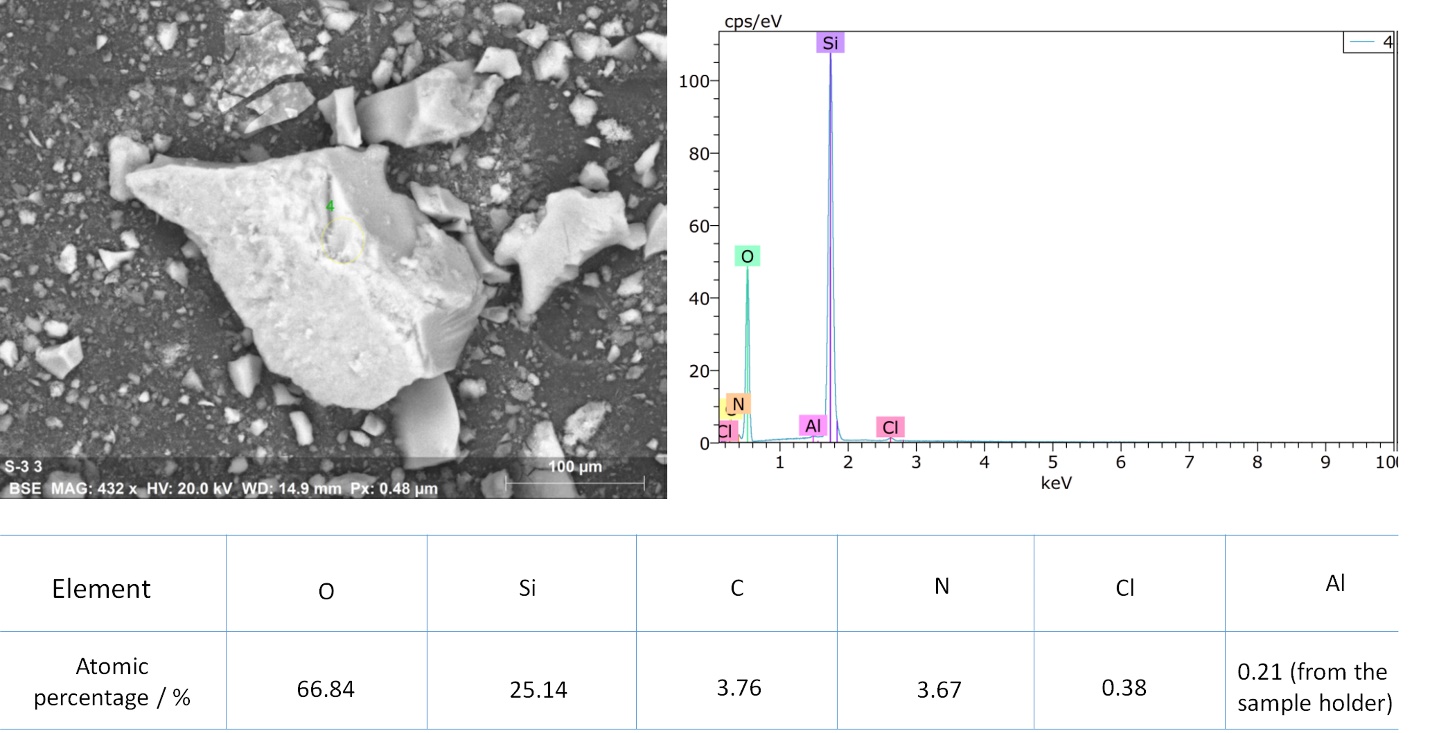


**Figure S7.** EDX analysis of the SiO_2_ nanoparticles.

**Figure S8.** FTIR spectra of pristine and calcinated SiO_2_ nanoparticles.

**Figure S9.** The relationship of f_peak_ with d when χ_m_ is zero.

**Figure S10.** The relationship of RL_peak_ with d when χ_m_ is zero.

**Figure S11.** The relationship of Δf_10_ with d when χ_m_ is zero.

**Figure S12.** Comparison of the 2D contour plots of the SiO_2_ nanoparticles with (A) a real nonzero χm and (B) a zero χm.


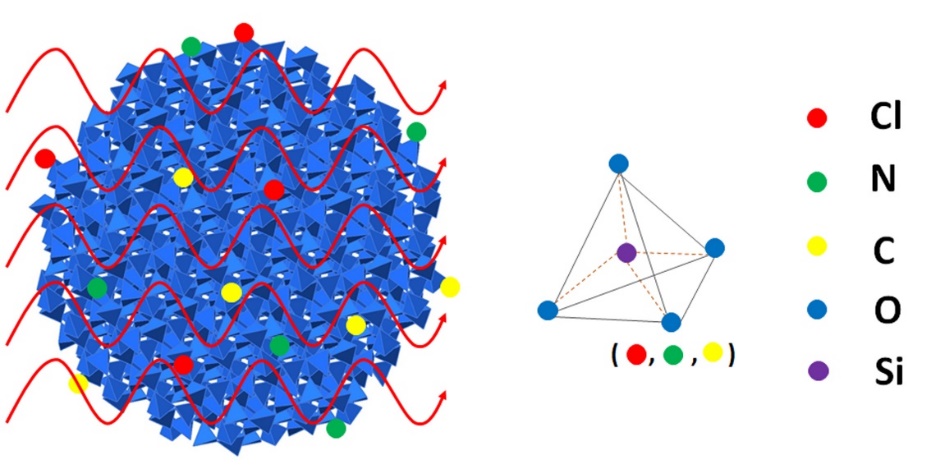


**Figure S13.** Illustration of the echoes of the microwave irradiation induced by the dipole rotations with heterogeneous atom incorporated conductive SiO_2_ nanoparticles.

**Figure S14.** Survey XPS spectrum of the SiO_2_ nanoparticles after calcination.

**Figure S15.** The O 1s core-level XPS spectrum of the SiO_2_ nanoparticles after calcination.

**Figure S16.** The Si 2p core-level XPS spectrum of the SiO_2_ nanoparticles after calcination.

**Figure S17.** The C 1s core-level XPS spectrum of the SiO_2_ nanoparticles after calcination.

**Figure S18.** Comparison of the permittivity of SiO_2_ nanoparticles before (calcinated SiO_2_) and after doping (doped SiO_2_): (A) real part, (B) imaginary part, and (C) electrical dissipation factor.

**Figure S19.** Comparison of the permeability of SiO_2_ nanoparticles before (calcinated SiO_2_) and after doping (doped SiO_2_): (A) real part, (B) imaginary part, and (C) magnetic dissipation factor.

**Table S1.** Microwave absorption performance of various materials

| **Materials** | **RL_max_ / dB** | **f / GHz** | **Δf_10_ / GHz** | **Ref.** |
| --- | --- | --- | --- | --- |
| Graphite Flakes | -25.3 | 14.5 | 2.9 | 4 |
| Graphene Foam | -28.2 | 12.3 | 8.9 | 5 |
| Ni-coated CNTs | -19.2 | 8.6 | 1.8 | 3 |
| CNT/Varnish | -24.3 | 15.4 | 5.2 | 6 |
| Porous CFs | -30.7 | 9.7 | 3.5 | 7 |
| polypyrrole/PVC | -46.8 | 13.6 | 4.9 | 8 |
| γ-Fe_2_O_3_ | -25.8 | 16.1 | 2.7 | 9 |
| Fe_3_O_4_ | -30.3 | 13.5 | 0.9 | 10 |
| α-MnO_2_ nanorod | -41.0 | 8.7 | 1.8 | 11 |
| ZnO | -37.0 | 6.2 | 2.1 | 12 |
| BaFe_12_O_19_/PANI | -13.0 | 7.8 | 0.5 | 13 |
| BaTiO_3_/PANI | -13.8 | 11.6 | 2.2 | 13 |
| Al_0.03_Si_99.97_C | -25.4 | 8.4 | 2 | 15 |
| SiC_1.09_N_0.82_O_0.22_ | -53.0 | 9.5 | 3 | 16 |
| Hydrogenated TiO_2_ nanoparticles | -48.0 | 9.6 | 15 | 17 |
| Hydrogenated TiO_2_ nanosheets | -37.9 | 13.9 | 8.5 | 18 |
| Part. Cryst. TiO_2_ | -36.9 | 14.3 | 1 | 20 |
| Hydrogenated ZnO | -38.0 | 15.2 | 14 | 2 |
| Hydrogenated BaTiO_3_ | -36.9 | 11.9 | 3.4 | 19 |
| SiO_2_:Cl: | -17.3 | 3.3 | 0.9 | 21 |
| SiO_2_-carbonyl iron | -21.5 | 12.2 | 4.3 | 22 |
| Fe-SiO_2_ | -55.0 | 5.3 | 2.8 | 23 |
| Conductive SiO_2_ nanoparticles | **-55.1** | **7.3** | **6.2** | **This work** |
